# Preoperative difference between 2D and 3D planning correlates with difference between planned and achieved surgical correction in patient‐specific instrumented total knee arthroplasty

**DOI:** 10.1002/jeo2.70128

**Published:** 2024-12-30

**Authors:** Patrick Pflüger, Alberto Pedrazzini, Lukas Jud, Lazaros Vlachopoulos, Sandro Hodel, Sandro F. Fucentese

**Affiliations:** ^1^ Department of Orthopedics, Balgrist University Hospital University of Zurich Zurich Switzerland

**Keywords:** computed tomography, computer‐assisted surgery, knee osteoarthritis, lower extremity, total knee arthroplasty

## Abstract

**Purpose:**

The goals of this study were (1) to assess whether the preoperative difference between modalities and extent of deformity are associated with a higher difference between planned and achieved surgical correction and (2) if they yield a higher probability of intraoperative adjustments.

**Methods:**

Retrospective single‐centre analysis of patients undergoing patient‐specific instrumented (PSI) total knee arthroplasty (TKA). Preoperative radiographic parameters were analysed on weightbearing (WB) long‐leg radiographs (LLR) and nonweightbearing (NWB) computed tomography (CT). The 2D/3D difference was calculated as the difference between preoperative WB‐LLR (2D) hip–knee–ankle angle (HKA), and NWB CT (3D) HKA. Surgical records were screened to retrieve intraoperative adjustments to the preoperative plan. Postoperative assessment was performed on WB LLR.

**Results:**

Two‐hundred‐eighty‐two knees of 263 patients were analysed. The difference of postoperative achieved to planned HKA (HKA_Difference_) was 2.2° ± 1.7°. The preoperative 2D HKA showed the highest correlation with HKA_Difference_ (*r* = −0.37, 95% confidence interval [CI]: −0.48 to −0.26, *p* < 0.001). Intraoperative adjustments were performed in 60% (*n* = 170) of all knees. Patients with a preoperative coronal deformity of >7.8° had 10.55 higher odds for an intraoperative coronal adjustment (95% CI: 4.60–24.20, *p* < 0.001).

**Conclusion:**

The extent of deformity is associated with residual coronal deformity following PSI‐TKA. Patients with extensive coronal malalignment may benefit from an adaptation of the preoperative surgical plan to avoid unintended postoperative coronal malalignment. Despite the advancements with 3D preoperative planning, intraoperative adjustments in PSI‐TKA are frequently performed, in particular in patients with a higher preoperative varus/valgus deformity.

**Level of Evidence:**

Level III.

**Abbreviations**
AUC: area under the curve
BMI: body mass index
CI: conventional instrumentation
CT: computed tomography
HKA: hip–knee–ankle angle
ICC: intraclass correlation coefficient
JLCA: joint line convergence angle
LLR: long‐leg radiographs
MRI: magnetic resonance image
NWB: nonweightbearing
PSI: patient‐specific instrumented
ROM: range of motion
TKA: total knee arthroplasty
WB: weightbearing

## INTRODUCTION

Patient‐specific instruments (PSI) for total knee arthroplasty (TKA) were introduced to improve reliability and accuracy of implant alignment and facilitate operating room procedures [11]. In comparison to surgery with conventional instrumentation (CI), PSI can improve the accuracy of component alignment and may reduce operative time and blood loss [25, 26, 27]. Despite these improvements, hip–knee–ankle axis (HKA) malalignment (deviation >3°) can be seen in up to 21% of patients following PSI‐TKA [26]. The causes for malalignment can be attributed to patient‐specific and surgeon‐specific factors and also seem to vary between different PSI systems [9, 21]. Implant malalignment can be a risk factor for poorer mid‐term patient‐reported outcome and long‐term implant failure [9, 12].

However, current literature advocates for more personalised alignment strategies in TKA and has led to discussions about traditional safe zones [23]. Independent of the alignment strategy, achieving the planned surgical correction is considered an important determinant of a favourable outcome [7, 16].

In PSI‐TKA, the 3D models and cutting jigs are based on a preoperative computed tomography (CT) scan or magnetic resonance image (MRI) [11]. Accordingly, image acquisition is performed in nonweightbearing (NWB) supine position, without loading forces across the knee joint, not affecting the lower limb alignment [1, 4, 8, 24, 29]. Studies demonstrated that the coronal alignment in preoperative 3D models for PSI‐TKA significantly differed from 2D weightbearing (WB) state and the difference between modalities correlated with the extent of coronal deformity [13, 18, 20]. The extent to which this influences the postoperative outcome in PSI‐TKA is unclear. For high tibial osteotomy, neglecting the WB state can lead to a potential under correction and therefore, studies try to incorporate the WB state in 3D planning [5, 6]. In unicompartmental knee arthroplasty, there were attempts to analyse risk factors for postoperative malalignment and a high arithmetic HKA was identified as risk factor for valgus malalignment [14, 15].

The goals of this study were (1) to assess whether the preoperative difference between modalities and the extent of preoperative deformity are associated with a higher difference between planned and achieved coronal surgical correction and (2) if they yield a higher probability for intraoperative adjustments. We hypothesised that the difference between 2D and 3D preoperative planning influences postoperative alignment and the probability for intraoperative adjustments.

## METHODS

### Study cohort

After ethical approval (2022‐02134), all consecutive patients that underwent surgery for a primary PSI‐TKA (Medacta SA, MyKnee GMK©) were screened between 2015 and 2020 (*n* = 332) from a single centre. Inclusion criteria comprised patients with complete preoperative knee x‐ray, NWB CT of the lower extremity, WB long‐leg radiographs (LLR) preoperative and 1 year after mechanically aligned PSI‐TKA, and a complete preoperative planning protocol in 2D and 3D.

After excluding patients, without complete preoperative planning information (*n* = 5), missing demographic information (*n* = 2), intraoperative conversion to other TKA model (*n* = 10), incomplete postoperative radiographs (*n* = 34), restricted kinematical alignment (*n* = 21), 282 knees of 263 patients were available for final analysis. Patients' medical records were reviewed, and the following data were obtained: age at surgery, gender and body mass index (BMI). Preoperative range of motion (ROM) was assessed and the passive extension deficit graded into four different stages (1: < 5°, 2: 5°‐10°, 3: 10°‐15°, 4: > 15°).

### Radiographic analysis

Two‐dimensional analysis of the original preoperative surgeon's planning of all patients was retrospectively reviewed and included routine radiographic assessment of WB LLR (EOS Imaging). For interrater reliability, the preoperative 2D deformity analysis was conducted again. One year following PSI‐TKA WB LLR were assessed by two raters, and the frontal LLR was imported to mediCAD® software (2D V7.0, Hectec GmbH) for further analysis. After manually selecting the centre of the femoral head, apex of the greater trochanter, femoral and tibial joint line, medial and lateral border of the femoral condyles and tibial plateau, medial and lateral border of the talus and the joint line of the talus, automatic deformity analysis is performed by the software according to Paley et al. [17]. The following radiographic parameters were assessed: HKA (°), mechanical medial proximal tibia angle (°), mechanical lateral distal femoral angle (°), joint line convergence angle (°; + = lateral opening, − = medial opening) [28].

Three‐dimensional analysis of preoperative planning was performed with a specific software (Medacta SA, MyKnee GMK©) based on preoperative NWB CT (planned HKA). The method of the preoperative 3D analysis was outlined in detail previously [20].

Patients were stratified according to the HKA (°) into three different groups: Varus = 2D HKA ≤ 78°, Neutral = 2D HKA > 178° < 182°, Valgus = 2D HKA ≥ 182°. The difference between preoperative 2D WB HKA and 3D NWB HKA was calculated (2D/3D difference) as described by Pflüger et al. [20]. To analyse whether the difference of 2D and 3D planning influences the postoperative coronal alignment three groups were formed: small 2D/3D difference (< 3°), intermediate 2D/3D difference (3°–5°) and high 2D/3D difference (>5°).

Intraoperative adjustments to the coronal alignment (varus/valgus) were included for the analysis of the difference between planned and postoperative 2D WB HKA as follows:

$${\text{HKA}}_{\text{Difference}}({}^{\circ})=(\text{Planned HKA}({}^{\circ})\pm \text{intraoperative coronal adjustments}({}^{\circ}))‐\;\text{post operative HKA}({}^{\circ}).$$

### Intraoperative adjustments

Operative records were screened to assess intraoperative deviations from the preoperative plan. The planned and definitive implants, as well as inlay type were assessed. To report the rate of intraoperative adjustments to the preoperative plan, all intraoperative osseous adjustments (coronal, sagittal, rotational) were recorded. For the analysis of the impact of preoperative 2D/3D HKA difference on intraoperatively performed coronal adjustments, only the coronal corrections were considered. The surgeon described which correction cutting jigs (varus/valgus) were used and the amount of intended coronal correction. Additional procedures (e.g., lateral patella lengthening, facetectomy) were recorded.

### Surgical technique

The MyKnee system (Medacta SA, GMK©) is based on preoperative NWB CT analysis. The 3D model is used to perform the deformity analysis, select the most suitable implant and create the cutting jigs for intraoperative use. According to the surgeons' preferences, the implant sizes and the planned alignment can be adjusted. The strategy was to achieve a mechanically aligned PSI‐TKA.

All patients underwent cemented cruciate sacrificing PSI‐TKA. Patella resurfacing was not routinely performed.

After visualisation of the joint, the tibial cut was set first. The patient‐specific cutting jig was referenced to the bony landmarks and alignment checked manually regarding coronal (alignment rod) and slope (angel wing) correction. Following the tibial cut, soft‐tissue laxity was assessed (extension balancing) with the help of the femoral cutting jig and if deemed sufficient, surgery was continued with the femur. Flexion balancing was performed with the help of the MyKnee rotating plate and MyKnee rotational guide. Depending on the surgeons' preference and intraoperative stability, adjustments were performed with the respective correction cutting jigs or by adjusting the rotation of the PSI as well as component sizes. The trial components were inserted to check the range of motion, stability in flexion and extension and patella tracking. If this was satisfactory, the definitive components were implanted. The arthrotomy and skin were sutured without the routine insertion of drainage.

All patients were allowed immediate WB with crutches for 4 weeks. To mitigate a potential bias due to surgeon's experience, surgeons were classified as low volume or high volume according to the numbers of total knee arthroplasties performed per year (high volume > 50 cases per year (Appendix: Table A1).

### Statistics

Data are presented as mean ± standard deviation or median and interquartile range. Rstudio (R version 4.4.0 [2024‐04‐24], PBC) was used for data processing and a *p *< 0.05 was considered statistically significant.

Data normality was assessed graphically (quantile‐quantile [Q‐Q] plot) and numerically (Shapiro–Wilk test).

To assess significant differences between the two groups, the two‐tailed independent *t* test or Mann–Whitney U test was used. For comparison of multiple groups, analysis of variance was performed with Tukey post hoc test.

We calculated Spearman's rank correlation coefficient to analyse the correlation of patient‐specific and radiographic factors with HKA_Difference_. A post hoc power calculation revealed an achieved power of 0.99 with the determined correlation of 0.37, α error of 0.05 and sample size of 282 (G*Power Version 3.1.9.6).

Performance analysis was conducted with radiographic factors that showed a significant correlation with HKA_Difference_, using receiver operating characteristic curve analysis to calculate the area under the curve (AUC) with the respective 95% confidence intervals (CIs). The optimal cut‐off value was determined at the maximum Youden index. Odds ratios for intraoperative adjustments were calculated with patients labelled either above or below the cut‐off value.

The preoperative and postoperative interrater reliability for the HKA was excellent (Intraclass correlation coefficient: ICC_preop_:1, 95% CI, 0.99–0.99, ICC_postop_: 0.97, 95% CI, 0.96–0.98).

## RESULTS

Included patients had a median age of 66 (14) years and 66% (*n* = 174) were female. The BMI was 31.2 (9.1) kg/m^2^ and a bilateral TKA was performed in 19 patients (7%). In total, the absolute difference between preoperative 2D and 3D HKA was 2.9 ± 2.4° (Table 1).

**Table 1:** Preoperative HKA (°) for weightbearing long‐leg radiographs and nonweightbearing CT (3D) as well as postoperative HKA.

	Varus (*n* = 178)	Neutral (*n* = 28)	Valgus (*n* = 76)	*p* Value
**Preoperative HKA (°)**				
2D	170.1 ± 4.4	179.8 ± 1.2	188.2 ± 4.3	<0.001
3D	173 ± 3.4	180 ± 1.7	185.6 ± 3.4	<0.001
Mean absolute difference	3.1 ± 2.1	0.9 ± 0.8	3.1 ± 3.0	<0.001
**Postoperative HKA (°)**				
2D	179 ± 2.9	181.5 ± 2.2	181.3 ± 2.7	<0.001
Absolute HKA_Difference_	2.2 ± 1.7	2 ± 1.8	2.2 ± 1.8	0.81

*Note*: The *p* value is stated for the comparison between the three groups. Post hoc testing confirmed the statistically significant differences between groups, except for the mean absolute difference of the preoperative HKA between varus and valgus knees (p_adjusted_ = 0.99) and postoperative HKA between valgus and neutral knees (p_adjusted_ = 0.92).

Abbreviations: CT, computed tomography; HKA, hip–knee–ankle angle.

### Postoperative outcome

**Figure 1:**
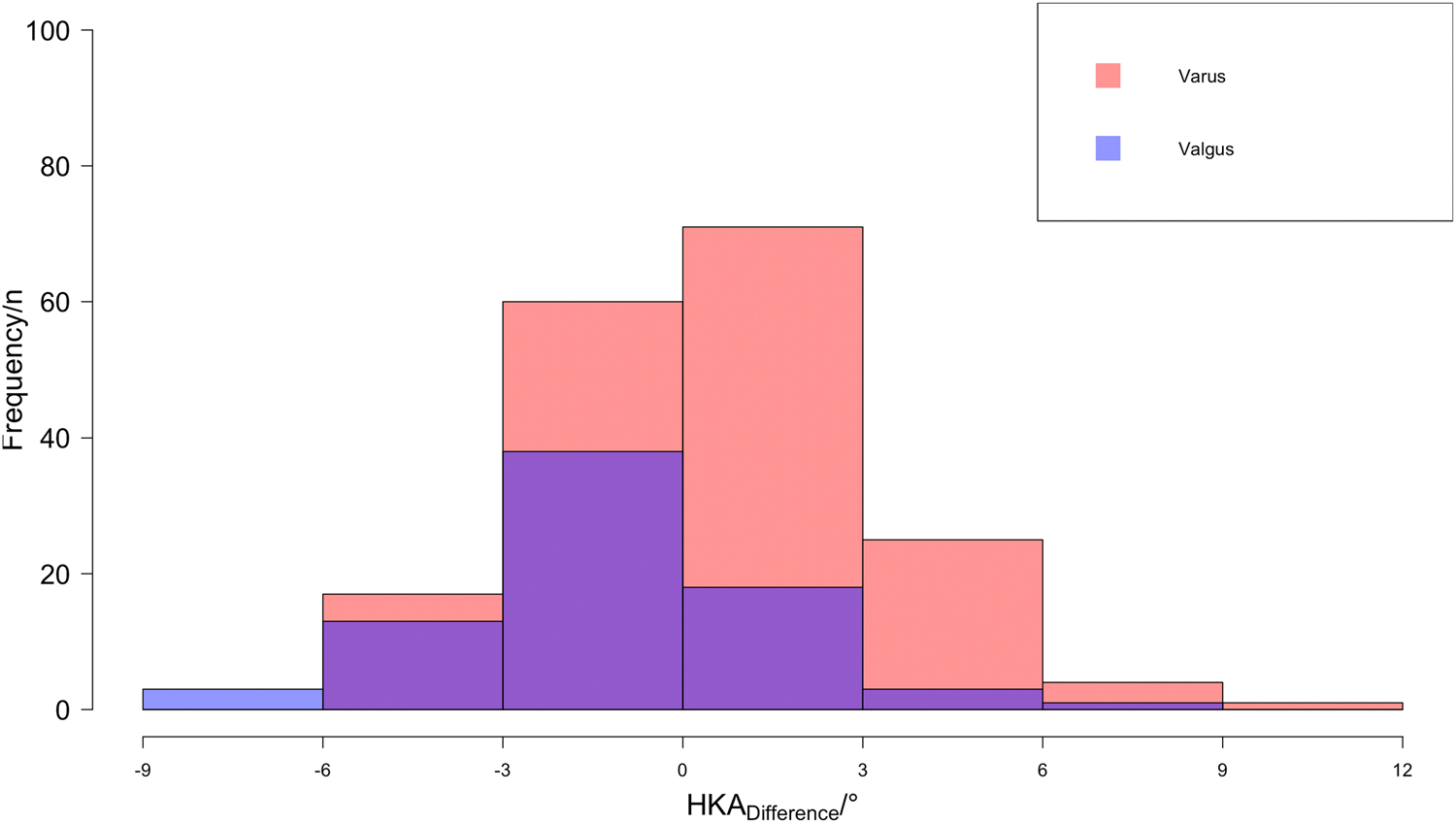
Histogram of the HKA_Difference_ for varus and valgus knees. HKA, hip–knee–ankle angle.

The absolute HKA_Difference_ for all operated lower limbs was 2.2° ± 1.7° (Table 1). The majority (76%, *n* = 214) had a postoperative absolute HKA_Difference_ of ≤3°, whereby preoperative valgus knees tend to remain in residual valgus and vice versa for varus knees (Figure 1). There were no statistically significant differences regarding the absolute HKA_Difference_ between the preoperative 2D/3D difference groups (*p* = 0.08).

The preoperative 2D HKA showed the highest correlation with HKA_Difference_ (ρ = 0.37, 95% CI, −0.48 to −0.26, *p *< 0.001) (Table 2). The absolute HKA_Difference_ neither significantly differed between patients with an intraoperative adjustment (2.2° vs. 2.1°, *p* = 0.49) nor an intraoperative coronal adjustment (2.2° vs. 2.2°, *p* = 0.99).

**Table 2:** Correlation of different preoperative parameters with HKA_Difference_.

Parameter	Spearman correlation coefficient (ρ, 95% CI)	*p* Value
BMI (kg/m^2^)	0.05 [−0.07; 0.16]	0.45
Preoperative ROM stage	−0.05 [−0.18; 0.08]	0.41
2D HKA (°)	−0.37 [−0.48; −0.26]	<0.001
3D HKA (°)	−0.36 [−0.46; −0.25]	<0.001
2D/3D difference (°)	−0.29 [−0.4; −0.18]	<0.001
JLCA (°)	−0.27 [−0.4; −0.17]	<0.001

Abbreviations: BMI, body mass index; CI, confidence interval; HKA, hip–knee–ankle angle; JLCA, joint line convergence angle; ROM, range of motion.

### Intraoperative adjustments

**Figure 2:**
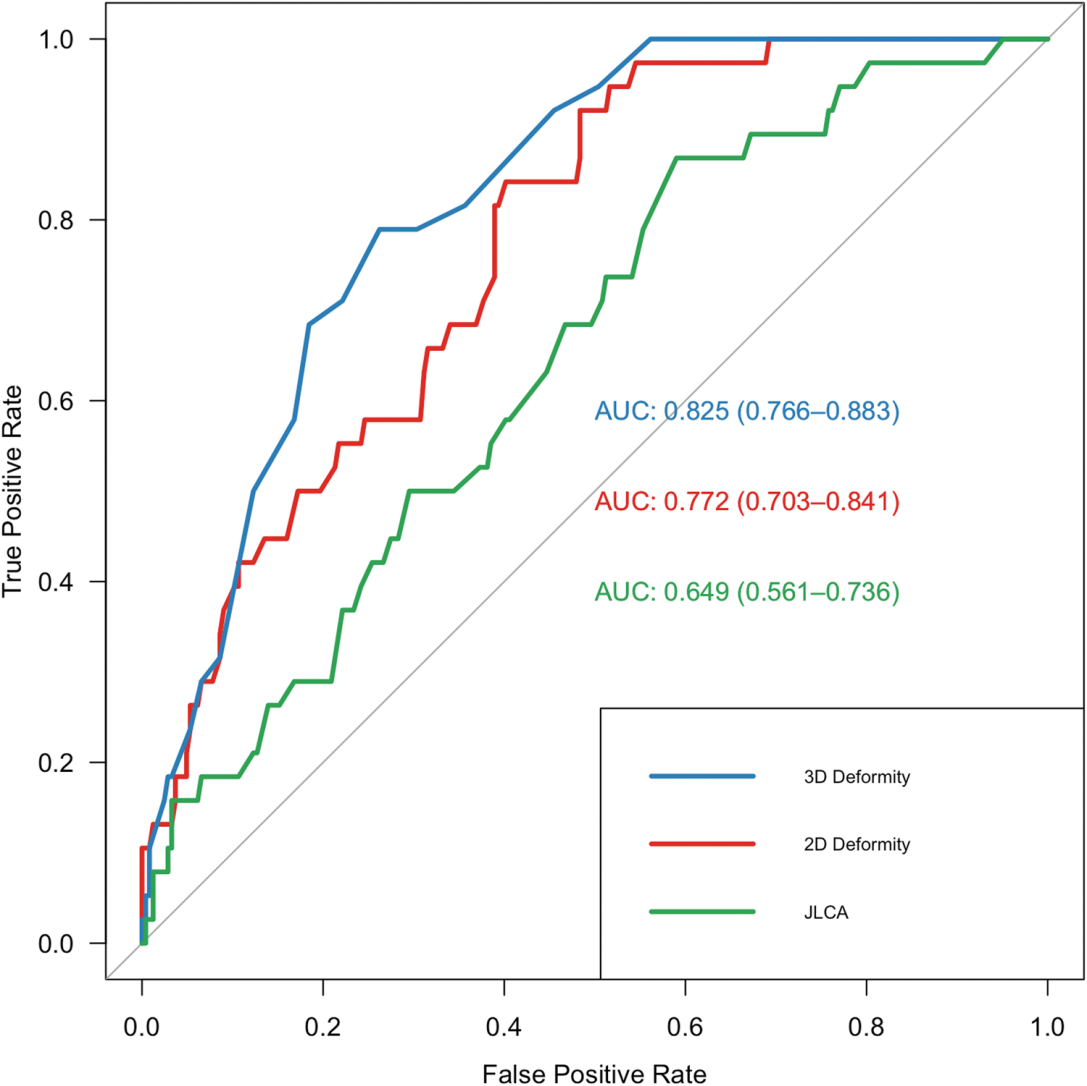
ROC curves of different preoperative radiographic parameters to predict an intraoperative coronal alignment adjustment. Only parameters with an area under the curve >0.6 are illustrated.

Intraoperative adjustments were performed in 60% (*n *= 170) of all knees. All osseous adjustments and those involving an adjustment of coronal alignment are illustrated in Table 3. In the high 2D/3D difference (>5°) group coronal adjustments were performed in 19% (*n *= 7), in the intermediate group in 18% (*n *= 12) and in the small group in 13% (*n *= 19) of all cases. Regarding diagnostic performance for predicting an intraoperative coronal adjustment, 3D coronal deformity showed the greatest AUC with 0.82 (95% CI, 0.77–0.88) (Figure 2). By identifying the greatest Youden index, a cut‐off value of 7.8° yielded a 0.79 sensitivity and 0.74 specificity to predict an intraoperative HKA adjustment (odds ratio: 10.55; 95% CI:4.60–24.20, *p* < 0.001).

**Table 3:** Intraoperative surgical adjustments stratified according to location of the adjustment.

	Varus (*n* = 178)	Neutral (*n* = 28)	Valgus (*n* = 76)
**Intraoperative adjustments**			
Femoral			
Coronal	7% (*n* = 13)	0% (*n* = 0)	8% (*n* = 7)
Rotational	11% (*n* = 19)	4% (*n* = 1)	11% (*n* = 9)
Tibial			
Coronal	12% (*n *= 21)	0% (*n* = 0)	4% (*n* = 3)
Sagittal	9% (*n* = 16)	11% (*n* = 3)	9% (*n* = 7)
**Total**	**60% (*n*** = **106)**	**46% (*n*** = **13)**	**67% (*n*** = **51)**

*Note*: In six knees, the intraoperative coronal adjustment was performed femoral and tibial. Total: knees with intraoperative adjustments (femoral and tibial) including adjustment of extension gap (proximalization/distalization without coronal adjustment). Change of implant size is not considered as femoral or tibial adjustment.

## DISCUSSION

The most important finding of this study is that the extent of coronal deformity is associated with a higher difference between planned and achieved coronal surgical correction following PSI‐TKA. Intraoperative adjustments were performed in more than half of all PSI‐TKAs, of which the coronal alignment was adjusted in one‐fifth. Patients with extensive coronal malalignment may benefit from adaptation of the preoperative surgical plan to avoid unintended postoperative coronal malalignment.

PSI can improve the accuracy of rotational alignment in TKA and can reduce the proportion of outliers from the target zone [25, 26, 27]. Tandogan et al. reported in their meta‐analysis, including six studies comparing CI to PSI, a lower rate of outliers using PSI [25]. Their results showed especially for the rotational alignment a favourable outcome. The rate of coronal malalignment differed significantly between the included studies, resulting in a substantial heterogeneity [25]. Thienpont et al. analysed a total of 44 studies, which included 2866 knees that underwent surgery with PSI and calculated a pooled risk for coronal malalignment >3° of 20.2% [26]. In order to reduce heterogeneity, Tibescu et al. conducted their systematic review of the outcome following one specific PSI‐TKA system. Analysing 25 studies using a specific MRI‐based PSI‐TKA system, they found an improved alignment accuracy (mechanical axis outlier rate: 14.1%) and surgical efficiency compared to CI [27].

Pauzenberger et al. investigated the outcome following mechanical aligned CT‐based PSI‐TKA in 815 cases. They reported a mean absolute deviation from targeted HKA of 1.7° with a HKA outlier rate (>3°) of 10.1% [19]. This is in line with our findings with a mean absolute HKA_Difference_ of 2.2°. Another study including 708 TKAs with the same CT‐based PSI system reported comparable results with an overall postoperative deviation of the targeted HKA of −1.2° and an HKA outlier rate (>3°) for the PSI group of 3% [3]. In a previous study involving the same surgeon, the HKA outlier rate following PSI‐TKA was substantially higher with 12.4% [10]. The authors, therefore, argue that the lower outlier rate in the more recent study might be contributed to a learning curve [3]. Contrary to this hypothesis, their previous study found no significant difference between junior and experienced surgeons regarding the outlier rate, and another study investigating the learning curve with PSI‐TKA was unable to detect a significant improvement with more cases [2, 10]. In our study, we also could not detect a difference in postoperative coronal alignment between low‐ and high‐volume knee surgeons (Appendix).

Taking together all studies and meta‐analysis, the range of postoperative coronal malalignment following PSI‐TKA shows a substantial heterogeneity and possible risk factors are not yet identified. More recent studies investigating the radiographic outcome of PSI‐TKA show a trend to lower postoperative malalignment, but the reasons are not fully understood. To elucidate possible factors influencing the difference between planned and achieved coronal surgical correction in PSI‐TKA, we conducted a thorough analysis of pre‐ and intraoperative risk factors.

Studies analysing the preoperative planning in PSI‐TKA demonstrated, that the coronal alignment of preoperative 3D models significantly differs from 2D assessment and the difference between modalities correlates with the extent of coronal deformity [13, 18, 20]. This might be due to the influence of WB state, since position‐dependent loading forces across the knee joint significantly alter lower limb alignment [1, 4, 8, 24, 29]. In the present study, the postoperative HKA_Difference_ did not show a significant difference for a higher preoperative 2D/3D HKA difference. But one needs to keep in mind that the preoperative 2D HKA showed the highest correlation with HKA_Difference_ and that the preoperative 2D/3D HKA difference correlates with the extent of coronal deformity [20]. In a mechanical aligned knee, the operative correction in severe varus/valgus deformity needs to be significantly greater, possibly contributing a higher probability for postoperative coronal malalignment in primary PSI‐TKA.

The literature regarding risk factors for postoperative malalignment in PSI‐TKA is scarce [26]. In knee‐preserving surgery, the influence of WB state was identified as possible risk factor for an under correction of coronal lower limb alignment following 3D‐based (PSI) high tibial osteotomy [5]. Therefore, studies try to incorporate the WB state in preoperative 3D planning [6, 22].

The preoperative 3D deformity showed the best diagnostic performance for predicting an intraoperative HKA adjustment. Patients with preoperative coronal deformity ≥7.8° showed a ninefold increased probability for an unplanned intraoperative coronal adjustment. An intraoperative HKA adjustment did not result in a significantly different HKA_Difference_ in comparison to cases without intraoperative coronal adjustment. Other studies utilising the same CT‐based PSI‐TKA system did not report intraoperative adjustments and their possible influence on postoperative malalignment [3, 19]. Koch et al. reported only intraoperative changes of the planned size of components in a total of 10.8% [10]. In the present study, the rate of component change was higher (Appendix, Table A2). Due to the limited data in the current literature, it is difficult to compare the findings.

The present study investigated a representative patient cohort and showed that in primary PSI‐TKA, the planned coronal alignment can be reliably achieved. Potential pre‐ and intraoperative factors contributing to a difference between planned and achieved coronal surgical correction were analysed. A higher preoperative coronal deformity correlates with the difference between planned and achieved HKA and poses a risk factor for an intraoperative coronal adjustment. Despite the advantages of the preoperative 3D model, intraoperative adjustments are performed in a substantial proportion of cases to ensure a balanced knee. However, these intraoperative adjustments do not significantly influence the difference between planned and achieved HKA.

A limitation of the study is its retrospective single‐centre design. Furthermore, the preoperative 3D planning was performed using a specific software by one manufacturer and based on manual determination of bony landmarks in preoperative NWB CT. Adjustments (component size, alignment) of the preoperative 3D planning was done by the treating surgeon. Intraoperative adjustments were performed depending on the intraoperative assessment of the surgeon. Patients were treated by different surgeons over a period of 5 years which leads to an undesired heterogeneity. However, to mitigate this potential bias, additional analysis showed no significant difference among the treating surgeons.

## CONCLUSION

The range of postoperative coronal malalignment following PSI‐TKA shows a substantial heterogeneity and possible risk factors are not yet fully understood. The present study showed that the extent of deformity is associated with residual coronal deformity following PSI‐TKA. Patients with extensive coronal malalignment may benefit from an adaptation of the preoperative surgical plan to avoid unintended postoperative coronal malalignment. Despite the advancements with 3D preoperative planning, intraoperative adjustments in PSI‐TKA are frequently performed, in particular, in patients with a higher preoperative coronal deformity.

## AUTHOR CONTRIBUTIONS

Patrick Pflüger carried out the data curation, statistical analysis and drafted the manuscript. Sandro Hodel participated in the design of the study and helped to draft the manuscript. Alberto Pedrazzini and Lukas Jud helped with data acquisition and processing. Lazaros Vlachopoulos and Sandro F. Fucentese conceived the study and participated in its design and coordination. All authors read and approved the final manuscript.

## CONFLICT OF INTEREST STATEMENT

Sandro F. Fucentese is a consultant for Medacta SA (Switzerland), Smith & Nephew (United Kingdom), Zimmer Biomet and Karl Storz SE & Co. KG (Germany). The remaining authors declare no conflict of interest.

## ETHICS STATEMENT

The study was conducted in accordance with the Declaration of Helsinki and approved by the local Ethics Committee (2022‐02134). Written informed consent was obtained from all patients.

## Data Availability

The data that support the findings of this study are available from the corresponding author upon reasonable request.

## Appendix

**Table A1:** Overview of cases per surgeon classified according to high volume and low volume with HKA_Difference_.

	High volume	Low volume	*p* Value
***Number of cases of the study cohort (n, percentage)***	177 (63%)	105 (37%)	‐
**Absolute HKA**_**Difference**_. (mean, SD**)**	2.1 ± 1.7	2.3 ± 1.7	0.56

*Note*: High volume surgeon ≥ 50 total knee arthroplasties per year.

Abbreviation: HKA, hip–knee–ankle angle.

**Table A2:** Intraoperative implant size changes according to location and proportion of inlays >10 mm.

Tibia implant size	Femur implant size	
	*Changed*	*Planned*
*Changed*	26% (*n *=72)	19% (*n *= 53)
*Inlay deviation*	9% (*n *= 25)	4% (*n *= 10)
*Planned*	24% (*n *= 68)	32% (*n *= 89)
*Inlay deviation*	10% (*n *= 28)	8% (*n *= 23)
